# Lean body mass index and hypertension risk in men: a nationwide epidemiological cohort study

**DOI:** 10.1038/s41440-025-02447-x

**Published:** 2025-11-10

**Authors:** Tatsuhiko Azegami, Hidehiro Kaneko, Akira Okada, Yuta Suzuki, Kazuki Aoyama, Katsuhito Fujiu, Norifumi Takeda, Hiroyuki Morita, Takashi Yokoo, Masaomi Nangaku, Koichi Node, Norihiko Takeda, Hideo Yasunaga, Kaori Hayashi

**Affiliations:** 1 Division of Nephrology, Endocrinology, and Metabolism, Department of Internal Medicine, Keio University School of Medicine, Tokyo, Japan; 2 Department of Cardiovascular Medicine, The University of Tokyo, Tokyo, Japan; 3 Department of Advanced Cardiology, The University of Tokyo, Tokyo, Japan; 4 Department of Prevention of Diabetes and Lifestyle-Related Diseases, Graduate School of Medicine, The University of Tokyo, Tokyo, Japan; 5 Center for Outcomes Research and Economic Evaluation for Health, National Institute of Public Health, Saitama, Japan; 6 Division of Nephrology and Hypertension, Department of Internal Medicine, Jikei University School of Medicine, Tokyo, Japan; 7 Division of Nephrology and Endocrinology, The University of Tokyo Graduate School of Medicine, Tokyo, Japan; 8 Department of Cardiovascular Medicine, Saga University, Saga, Japan; 9 Department of Clinical Epidemiology and Health Economics, School of Public Health, The University of Tokyo, Tokyo, Japan

**Keywords:** Lean mass, Hypertension, Database

## Abstract

Hypertension, a leading global health challenge, is intricately linked to obesity in its pathogenesis. Body mass index, a common indicator of obesity, cannot distinguish between fat mass and lean body mass, which exert contrasting cardiovascular effects. This study aimed to evaluate the lean body mass index (LBMI), derived from height, weight, and waist circumference, as a predictor of hypertension risk in men. This retrospective study utilized a large-scale real-world database to evaluate the association between LBMI and hypertension risk in men. Hypertension incidence was identified via ICD-10 codes (I10–I15) utilizing an administrative claims database. Cox regression and spline models assessed risk, adjusting for confounders. To confirm the robustness of findings, stratified and sensitivity analyses were also conducted. Among 384,551 men (median age 51 years), lower quartile in LBMI was associated with a higher risk of hypertension onset in multivariable Cox regression (hazard ratio [95% confidence interval]: Q1, 1.20 [1.15–1.26]; Q2, 1.06 [1.02–1.10]; Q3, 1.03 [0.99–1.06]; Q4, 1 [reference value]). In the restricted cubic spline regression model, the risk of hypertension increased as LBMI decreased. Consistent results were observed across stratified analyses, including older adults and non-obese individuals, and the reliability of the findings was confirmed through sensitivity analyses such as multiple imputation and competing risks analysis. In conclusion, lower LBMI was associated with a higher risk of hypertension in men, underscoring the importance of promoting lean body mass. Future research should explore whether increasing lean body mass could reduce hypertension incidence and its complications.

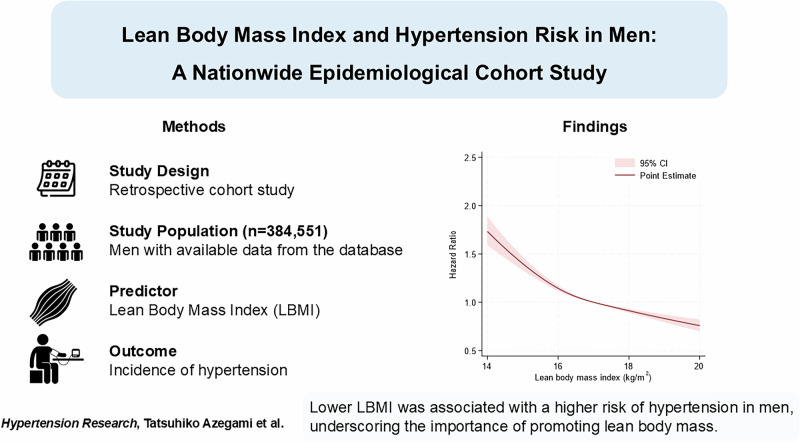

## Introduction

Hypertension, with its high prevalence and role as a major risk factor for cardiovascular diseases and chronic kidney disease, is recognized as one of the most critical global public health challenges [1]. Therefore, accurately identifying individuals at risk of developing hypertension through population-level health screenings is an essential medical task. Among the key risk factors, obesity is strongly associated with hypertension [2, 3]; however, body mass index (BMI), a widely used measure of obesity, cannot clearly differentiate between fat mass and lean body mass (LBM), which may have distinct effects on cardiovascular health [4]. Fat mass is closely linked to the onset and progression of hypertension, metabolic syndrome, and cardiovascular diseases [2, 4, 5]. In contrast, lean body mass, composed primarily of skeletal muscle and organ tissues, may play a protective role in regulating metabolism and hemodynamics [4, 5], making it a subject of increasing interest in recent research.

Accurately measuring LBM has traditionally required advanced imaging techniques such as dual-energy X-ray absorptiometry (DXA) [6]. However, the high costs and logistical challenges associated with these imaging-based assessments have significantly limited their applicability in routine health screenings. To overcome this limitation, a recent study has proposed a simple predictive formula for estimating LBM in men based on age, height, weight, waist circumference, and race [7]. Notably, low and high LBM have been identified as significant risk factors for increased mortality among men [7]. Building on this foundation, our study utilized an administrative claims database to evaluate the association between lean body mass index (LBMI)–calculated using a simplified estimation formula based on easily obtainable anthropometric data from health screenings–and the risk of developing hypertension. This approach aims to provide a practical and scalable method for assessing LBM in large populations, contributing to improved risk stratification and preventative healthcare strategies.

## Methods

### Study design and data source

This retrospective observational study utilized the DeSC database (DeSC Healthcare Inc., Tokyo, Japan) form April 2014 to November 2022. The DeSC database contains individual health insurance claims for both inpatients and outpatients, coded according to the International Classification of Diseases, Tenth Revision (ICD-10), as well as annual health checkup records. These records include height, weight, waist circumference, blood pressure measurements, fasting laboratory data, and responses to a questionnaire regarding medical history, lifestyle, and current medications. The database encompasses three major health insurance systems in Japan: the health insurance for employees of large companies (Kempo), the National Health Insurance for nonemployees (Kokuho), and the Advanced Elderly Medical Service System for individuals aged ≥75 years (Koki Koreisha Iryo Seido). This coverage spans a wide age range, from young adults to the older adults, thereby ensuring a population representativeness.

### Ethics statement

The present study was approved by the Ethics Committee of the University of Tokyo (approval number: 2021010NI) and conducted according to the Declaration of Helsinki. The requirement for informed consent was waived because all data recorded in the DeSC database were anonymized and deidentified.

### Study participants

**Fig. 1:**
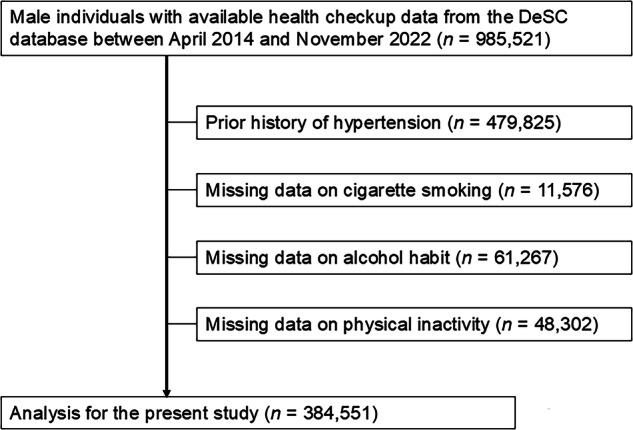
Flowchart Showing the Participant Selection Process. We extracted data for 985,521 male individuals with available data on health checkup enrolled in the DeSC Database between April 2014 and November 2022. Individuals with a prior history of hypertension (*n* = 479,825), and those with missing information on smoking status (*n* = 11,576), alcohol consumption (*n* = 61,267), or physical inactivity (*n* = 48,302) were excluded. Finally, we analyzed 384,551 men in the current study

Records of men with available health checkup data were selected (*n* = 985,521). Exclusions were made for those with a prior history of hypertension, defined as a diagnosis of hypertension based on ICD-10 codes, systolic blood pressure ≥140 mmHg, diastolic blood pressure ≥90 mmHg, or the use of antihypertensive medications (*n* = 479,825). Of the initial study population, individuals with missing information on smoking status (*n* = 11,576), alcohol consumption (*n* = 61,267), or physical inactivity (*n* = 48,302) were also excluded from the primary analyses to ensure interpretability and transparency of baseline characteristics and covariate adjustment. The flowchart illustrating the selection criteria is shown in Fig. 1. Finally, 384,551 men were included in the analysis.

### Measurements and definition

Measurement data from health checkups, including height, weight, waist circumference, blood pressure, lipids, and HbA1c, were retrieved from the DeSC database. Information on smoking status (current or non-current), alcohol consumption (daily or not daily), and physical inactivity (inactive or active) was also collected through a self-reported questionnaire completed during the health checkup.

The Japanese Ministry of Health, Labour and Welfare has provided a recommended protocol for blood pressure measurement. According to this protocol, healthcare professionals are advised to measure blood pressure on the right arm using either a standard sphygmomanometer or an automated device, after the individual has been seated at rest for five minutes. The measurements are performed 2 times, and the mean of the first and second readings should be recorded [8]. While these examinations are conducted based on national recommendations provided by the Ministry of Health, Labour and Welfare, actual measurement procedures may vary across facilities.

Obesity was defined as a BMI of ≥25 kg/m^2^. Diabetes was identified by an HbA1c level of ≥6.5% or the use of blood glucose-lowering medications. Dyslipidemia was diagnosed using the following criteria: a low-density lipoprotein cholesterol level of ≥140 mg/dL, a high-density lipoprotein cholesterol level of <40 mg/dL, a triglyceride level of ≥150 mg/dL, or the use of lipid-lowering medications. Data on smoking status (current or non-current), alcohol consumption (daily or not daily), and physical inactivity (inactive or active) were self-reported.

LBM and LBMI were calculated using the following formula based on previous studies [7, 9].

$${{\rm{LBM}}}\left({{\rm{kg}}}\right)=	19.363+0.001\times {{\rm{age}}}\left({{\rm{years}}}\right) +0.064\times {{\rm{height}}}\left({{\rm{cm}}}\right) \\ 	+ 0.756 \times {{\rm{body}}}\; {{\rm{weight}}} \left({{\rm{kg}}}\right) - 0.366 \times {{\rm{waist}}}\; {{\rm{circumference}}} \left({{\rm{cm}}}\right) \\ 	- 0.066 \times {{\rm{Mexican}}}[1\,{{\rm{if}}}\; {{\rm{yes;}}} \, 0\, {{\rm{if}}}\; {{\rm{no}}}] + 0.231 \times {{\rm{Hispanic}}}\,[1\,{{\rm{if}}}\; {{\rm{yes;}}}\,0\,{{\rm{if}}}\; {{\rm{no}}}] \\ 	+ 0.432 \times {{\rm{Black}}}[1\,{{\rm{if}}}\; {{\rm{yes;}}}\,0\,{{\rm{if}}}\; {{\rm{no}}}] - 1.007 \times {{\rm{Other}}}\; {{\rm{ethnicity}}}[1\,{{\rm{if}}}\; {{\rm{yes;}}} \,0 \,{{\rm{if}}}\; {{\rm{no}}}]$$

$${{\rm{LBMI}}}\left({{\rm{kg}}}/{{{\rm{m}}}}^{2}\right)={{\rm{LBM}}}({{\rm{kg}}})/{\left[{{\rm{h}}}{{\rm{eig}}}{{\rm{h}}}{{\rm{t}}}({{\rm{m}}})\right]}^{2}$$

### Outcome

The primary outcome was the incidence of hypertension (ICD-10 codes I10–I15).

### Statistical analysis

Continuous variables were presented as the median (interquartile range), and categorical variables were presented as numbers (percentages). The LBMI was divided into quartiles (Q1–Q4), and comparisons were made between each group. Cox regression analyses were conducted to assess the association between the LBMI and the subsequent risk of hypertension. Hazard ratios (HRs) and 95% confidence interval (CI) for the incidence of hypertension were calculated in an unadjusted model and after adjusting for potential confounders, including age, BMI, systolic and diastolic blood pressures, diabetes, dyslipidemia, cigarette smoking, alcohol drinking, and physical inactivity. In the restricted cubic spline regression model, the association between continuous changes in LBMI and the risk of incident hypertension was also evaluated after adjustment for age, BMI, systolic and diastolic blood pressures, diabetes, dyslipidemia, cigarette smoking, alcohol drinking, and physical inactivity. Four cutoff points for the LBMI (5, 35, 65, and 95 percentiles) were applied, with 17.0 kg/m^2^ set as the reference point.

To further explore the role of BMI, we similarly categorized BMI into quartiles (Q1–Q4) and evaluated its association with incident hypertension using Cox regression analysis. In addition, to compare the predictive value of LBMI and BMI, we constructed three multivariable models: one including LBMI categories only, one including BMI categories only, and one including both LBMI and BMI categories. We then calculated model performance metrics, including C-statistics, Akaike Information Criterion (AIC), and Bayesian Information Criterion (BIC), to assess and compare the discriminatory power of each model.

We conducted stratified and sensitivity analyses to confirm the robustness of the primary findings. We performed a subgroup analysis stratified by age (<65 or ≥65 years) and conducted a separate analysis focusing on non-obese individuals. We redefined hypertension incidence as cases where both ICD-10 code for hypertension and new prescriptions for anti-hypertensive medications, defined by WHO-ATC codes (C02, C03, C04, C07, C08, and C09), were present, and performed sensitivity analyses. We employed multiple imputations with chained equations to replace missing variables (smoking status, alcohol consumption, and physical inactivity). Additionally, we performed the Fine-Gray subdistribution hazard model for competing risks analysis, as death should be considered a competing risk with hypertension incidence.

Statistical analyses were performed using STATA version 18.0 (StataCorp LLC, College Station, TX, USA). A *P*-value of <0.05 was considered statistically significant.

## Results

Table 1 summarizes the clinical characteristics of the study participants. The median age (interquartile range) was 51 (41–65) years. The median LBM and LBMI were 48.6 (45.2–52.4) kg and 16.9 (16.1–17.9) kg/m^2^, respectively. Additionally, the median BMI was 22.8 (20.9–24.8) kg/m^2^. Systolic and diastolic pressures were 120 (111–128) mmHg and 73 (67–80) mmHg. Diabetes was observed in 6.9% of the study participants, and dyslipidemia in 48.6%.

**Table 1:** Baseline characteristics of the study participants

	Overall	Q1	Q2	Q3	Q4	*P* value
	*N* = 384,551	*N* = 96,138	*N* = 96,140	*N* = 96,136	*N* = 96,137	
Age (years), median (IQR)	51 (41–65)	57 (42–67)	53 (42–66)	51 (42–64)	48 (41–59)	<0.001
Lean body mass index (kg/m^2^), median (IQR)	16.9 (16.1–17.9)	15.5 (15.0–15.8)	16.5 (16.3–16.7)	17.4 (17.1–17.6)	18.7 (18.3–19.5)	<0.001
Lean body mass (kg), median (IQR)	48.6 (45.3–52.4)	44.3 (41.8–46.8)	47.4 (45.0–49.8)	49.8 (47.3–52.3)	54.3 (51.2–57.8)	<0.001
Body mass index (kg/m^2^), median (IQR)	22.8 (20.9–24.8)	19.7 (18.7–20.7)	21.9 (21.1–22.7)	23.6 (22.8–24.4)	26.4 (25.2–28.1)	<0.001
Obesity, *n* (%)	90,662 (23.6%)	28 (0.0%)	559 (0.6%)	12,565 (13.1%)	77,510 (80.6%)	<0.001
Systolic BP (mmHg), median (IQR)	120 (111–128)	117 (108–126)	119 (110–127)	120 (112–128)	122 (115–129)	<0.001
Diastolic BP (mmHg), median (IQR)	73 (67–80)	71 (65–78)	72 (66–79)	74 (67–80)	76 (70–81)	<0.001
Comorbidities, *n* (%)						
Diabetes	26,562 (6.9%)	6078 (6.3%)	5649 (5.9%)	6193 (6.4%)	8642 (9.0%)	<0.001
Dyslipidemia	186,985 (48.6%)	34,015 (35.4%)	42,453 (44.2%)	49,646 (51.6%)	60,871 (63.3%)	<0.001
Medical history, *n* (%)						
Myocardial infarction	383 (0.1%)	111 (0.1%)	86 (0.1%)	94 (0.1%)	92 (0.1%)	0.31
Heart failure	4,532 (1.2%)	1,429 (1.5%)	1,138 (1.2%)	1,036 (1.1%)	929 (1.0%)	<0.001
Stroke	4,099 (1.1%)	1,274 (1.3%)	1,021 (1.1%)	993 (1.0%)	811 (0.8%)	<0.001
Cigarette smoking, *n* (%)	118,001 (30.7%)	31,087 (32.3%)	27,839 (29.0%)	28,181 (29.3%)	30,894 (32.1%)	<0.001
Alcohol consumption, *n* (%)	116,437 (30.3%)	31,453 (32.7%)	31,181 (32.4%)	29,865 (31.1%)	23,938 (24.9%)	<0.001
Physical inactivity, *n* (%)	170,736 (44.4%)	43,465 (45.2%)	41,480 (43.1%)	41,747 (43.4%)	44,044 (45.8%)	<0.001
HbA1c (%), median (IQR)	5.5 (5.2–5.7)	5.4 (5.2–5.7)	5.4 (5.2–5.7)	5.5 (5.3–5.7)	5.5 (5.3–5.8)	<0.001
LDL cholesterol (mg/dL), median (IQR)	121 (101–143)	113 (94–135)	120 (100–141)	124 (104–145)	128 (109–149)	<0.001
HDL cholesterol (mg/dL), median (IQR)	56 (48–67)	62 (53–73)	58 (50–69)	55 (47–65)	51 (44–59)	<0.001
Triglycerides (mg/dL), median (IQR)	97 (68–144)	81 (59–116)	90 (65–131)	101 (71–148)	122 (84–180)	<0.001

Data are expressed as median (interquartile range) or number (percentage). Participants were divided into four groups based on the quartiles of the lean body mass index (Q1–Q4)

*IQR* interquartile range, *BP* blood pressure, *LDL* low-density lipoprotein, *HDL* high-density lipoprotein

**Fig. 2:**
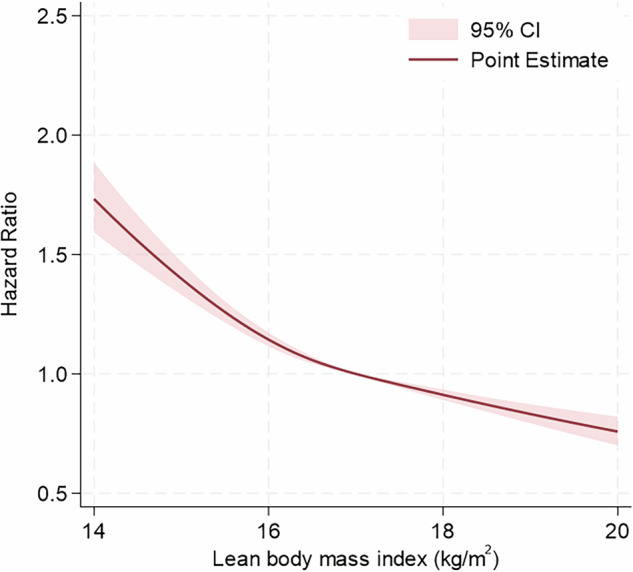
Restricted cubic spline showing hazard ratios for the incidence of hypertension along with lean body mass index. Restricted cubic spline with four knots (5, 35, 65, and 95 percentiles) shows the association of lean body mass index with the incidence of hypertension. Hazard ratios are adjusted for age, body mass index, systolic and diastolic blood pressures, diabetes, dyslipidemia, cigarette smoking, alcohol drinking, and physical inactivity. Lean body mass index of 17.0 kg/m^2^ was set as the reference point

During a median (interquartile range) follow-up of 1393 (683–2145) days, 40,312 cases (10.5%) of hypertension event were documented. Multivariable Cox regression analyses demonstrated that lower in LBMI (Q1–Q4) was associated with a higher risk of hypertension onset (HR [95% CI]: Q1, 1.20 [1.15–1.26]; Q2, 1.06 [1.02–1.10]; Q3, 1.03 [0.99–1.06]; Q4, 1 [reference value]) (Table 2). In the restricted cubic spline regression model, the risk of hypertension increased with the reduction in LBMI (Fig. 2). In contrast, higher BMI was associated with an increased risk of hypertension (HR [95% CI]: Q1, 1 [reference value]; Q2, 1.10 [1.07–1.14]; Q3, 1.20 [1.17–1.25]; Q4, 1.50 [1.45–1.56]) (Supplementary Table S1). To assess potential collinearity, we calculated the variance inflation factor (VIF) for key variables: 1.74 between systolic and diastolic blood pressure, 2.06 between continuous LBMI and BMI, 1.64 between LBMI categories and BMI, and 1.11 between LBMI categories and age, indicating no evident multicollinearity. The C-statistics for the multivariable model using LBMI alone and BMI alone were 0.7138 and 0.7148, respectively, with minimal improvement when both indices were included simultaneously (0.7149). The AIC/BIC values were 965334.1/965453.6 for LBMI, 965060.7/965180.2 for BMI, and 965003.8/965155.8 when both indices were included.

**Table 2:** Association between lean body mass index and hypertension onset: event frequency, incidence rates, and hazard ratios

	Number	Event	Incidence Rate (95% CI)	Model 1 (Unadjusted)	Model 2	Model 3
Q1	96,138	8926	243.7 (238.7–248.8)	0.74 (0.72–0.76)	1.25 (1.19–1.31)	1.20 (1.15–1.26)
Q2	96,140	9234	245.3 (240.4–250.4)	0.75 (0.73–0.77)	1.08 (1.04–1.12)	1.06 (1.02–1.10)
Q3	96,136	10,162	271.8 (266.5–277.1)	0.83 (0.81–0.85)	1.04 (1.01–1.07)	1.03 (0.99–1.06)
Q4	96,137	11,990	328.0 (322.1–333.9)	1 [Reference]	1 [Reference]	1 [Reference]

The incidence rate was per 10,000 person-years. Unadjusted and adjusted hazard ratios (95% CI) for the development of hypertension are shown. Model 1 is unadjusted. Model 2 includes adjustment for age, systolic and diastolic blood pressures, and body mass index. Model 3 includes adjustment for age, systolic and diastolic blood pressures, body mass index, diabetes, dyslipidemia, cigarette smoking, alcohol drinking, and physical inactivity

*CI* confidence interval

We conducted stratified and sensitivity analyses. First, we conducted a subgroup analysis stratified by age (<65 or ≥65 years). In multivariable Cox regression analyses, HR (95% CI) of the reduced LBMI (Q1) for the incidence of hypertension was 1.19 (1.12–1.26) and 1.14 (1.05–1.24) in individuals aged <65 and those aged ≥65 years, respectively (Supplementary Table S2). Second, a separate analysis focusing on non-obese individuals revealed that the HR (95% CI) of the decrease in LBMI (Q1) for hypertension incidence was 1.17 (1.09–1.26) (Supplementary Table S3). Third, we redefined hypertension incidence as cases where both the ICD-10 code for hypertension and new prescriptions for antihypertensive medications. Among individuals diagnosed with hypertension based on ICD-10 codes, 14,197 cases without antihypertensive medication prescriptions within six months after diagnosis were excluded, leaving 370,354 individuals for analysis. Low LBMI (Q1) was associated with higher risk of developing hypertension, with the HR (95% CI) of 1.25 (1.18–1.32) (Supplementary Table S4). Fourth, we employed multiple imputations with chained equations to replace missing variables and conducted an analysis involving 505,696 individuals. The presence of reduced LBMI (Q1) was a significant risk for the incidence of hypertension in this sensitivity analysis (HR [95% CI], 1.19 [1.14–1.24]) (Supplementary Table S5). Fifth, in the competing risks analysis with death as a competing event, we found the increased risk of lower LBMI (Q1) for hypertension with HR 1.23 (95% CI 1.15–1.32) (Supplementary Table S6).

## Discussion

Using a large-scale epidemiological real-world dataset, we analyzed 384,551 men without a history of hypertension who underwent annual health check-ups. Our analysis revealed a significant monotonic inverse association between LBMI and the risk of developing hypertension, with lower LBMI associating with a higher risk of hypertension. The robustness of our primary findings was strengthened by conducting stratified and sensitivity analyses.

We provided evidence supporting the crucial role of LBM in metabolic health and cardiovascular risk. Although BMI is a widely used indicator of obesity [10], it cannot distinguish between fat mass and lean mass, which may limit its interpretive accuracy in certain populations —particularly relevant in men, who generally have higher lean mass [11]. Moreover, in older adults, high BMI has occasionally been associated with better outcomes, a phenomenon known as the “obesity paradox.” [12] The reversal of the association between LBMI and hypertension after multivariable adjustment was largely explained by the inclusion of BMI in the model. This suggests that BMI, which is strongly correlated with both lean and fat mass, accounted for much of the observed change in the direction of the association. In our comparative analysis, BMI showed consistent associations with hypertension risk, especially at higher levels. Although LBMI had a smaller impact than BMI, it was independently associated with the risk of developing hypertension in multivariable models. Furthermore, even in the population with a BMI below 25 kg/m²—generally considered at lower risk for developing hypertension—low LBMI was associated with an increased risk. The addition of LBMI enabled more accurate stratification of hypertension risk, indicating that LBMI may provide complementary information in situations where BMI alone is insufficient.

While LBM can be accurately measured using imaging techniques such as DXA, these methods are impractical for large-scale hypertension risk screening. To address this issue, we used a previously established predictive formula for LBM based on height, weight, and waist circumference, which demonstrated high agreement with DXA measurements (R² = 0.91) [7]. We restricted our analysis to men because the LBM prediction formula was validated in male populations, in whom low and high LBM has been identified as a significant risk factor for increased mortality [7]. Since LBM is influenced by height, it was divided by the square of height to derive LBMI. Using this simple and non-invasive method, we successfully demonstrated an inverse association between LBMI and the risk of developing hypertension in a large-scale real-world dataset.

Several mechanisms may explain the protective effect of LBM on hypertension risk. First, skeletal muscle mass, a key component of LBM, improves glucose and lipid metabolism [13, 14], which may exert protective effects against atherosclerosis and contribute to maintaining blood pressure homeostasis. Additionally, low muscle mass may lead to an overestimation of creatinine-based eGFR [15], and even when the apparent eGFR is the same, individuals with lower muscle mass may have lower actual kidney function, which may in turn reduce the ability of the kidneys to adequately contribute to blood pressure regulation. Furthermore, a reduction in muscle mass may lead to an increase peripheral vascular resistance [16], thereby contributing to elevated blood pressure. Moreover, skeletal muscle functions as an endocrine organ, secreting myokines such as irisin, which exert anti-inflammatory effects and may play a role in vascular protection [17, 18]. These mechanisms support the hypothesis that higher LBM contributes to improved blood pressure regulation, as reflected in our findings.

Previous studies examining the association between LBM and hypertension have yielded inconsistent results. For example, a recent cross-sectional study using a nationally representative dataset in the United States reported a U-shaped association between LBM and hypertension risk, suggesting that both low and high levels of LBM may confer increased risk [19]. In that study, restricted cubic spline regression and piecewise linear models identified an inflection point at ~43.2 kg, below which LBM was negatively associated with hypertension, and above which it showed a positive association. This discrepancy with our findings, which demonstrated a monotonic inverse association, may be partially explained by population differences. Compared to Japan, the U.S. population includes a higher proportion of individuals with obesity, which may influence the association through greater lean mass driven by overall body size. Furthermore, body composition profiles and fat distribution differ substantially between ethnicities, which could contribute to the non-linear relationship observed in Western populations. Our findings suggest that, particularly in leaner populations such as Japanese men, lower LBM may play a more central role in the development of hypertension.

The present study has several clinical implications. BMI alone is insufficient to distinguish between fat mass and lean mass, but the use of LBMI allows for this distinction, helping to avoid both overestimation and underestimation of hypertension risk. While further investigation is needed, especially in older populations where muscle loss is common, maintaining or promoting LBM may be important for hypertension prevention. Therefore, public health policies should focus on supporting the promotion of LBM through physical activity and dietary measures, which could help reduce the global burden of hypertension and its related comorbidities.

The strengths of our study lie in the adoption of a simplified LBMI calculation method and the use of a large-scale real-world database to demonstrate the utility of LBMI for hypertension risk screening. Additionally, consistent results were observed across stratified analyses, including older adults and non-obese individuals (BMI <25 kg/m²), and the robustness and reliability of the findings were confirmed through sensitivity analyses such as multiple imputation and competing risks analysis. Furthermore, it is noteworthy that in our study, LBMI showed a significant association with the risk of incident hypertension independently of BMI. This suggests that LBMI may capture a distinct aspect of body composition and add predictive value, especially in individuals with lower BMI. While BMI remains a useful indicator overall, the combined use of BMI and LBMI may enhance hypertension risk stratification, particularly in populations with lower body mass.

However, several limitations must be acknowledged. First, the simplified LBM formula is practical and accounts for racial differences, including Mexican, Hispanic, Black, other groups [7, 9]. However, the “other” race constitutes only 3.9% of the total population (*n* = 5239), leaving the applicability of this formula to Asians uncertain, and the applicability of our findings to different ethnic groups and regions has not been validated.

Second, as our study is observational, causal inferences cannot be definitively made. Additionally, while our results from the multivariate analyses suggest that LBM plays a protective role against hypertension, the possibility of residual confounding factors, including kidney function and serum uric acid levels, cannot be entirely excluded. As shown in Table 1, baseline clinical characteristics differed between the groups, and therefore, differences in treatments administered during the observational period may also have influenced the risk of developing hypertension. However, detailed information regarding treatment specifics (such as medication dosages) and control status for comorbidities including diabetes and dyslipidemia were not available in the database, making further adjustment difficult. Thus, residual confounding related to treatment effects may still have impacted our results. In addition, individuals with low BMI and low waist circumference might represent a more frail population or those with undiagnosed cachexia, including underlying cancer or malnutrition. These conditions may lower blood pressure through mechanisms unrelated to muscle mass per se, potentially affecting our findings. Further studies incorporating nutritional status or comorbidity indices are warranted.

Third, reliance on administrative and self-reported data introduces the potential for measurement bias, particularly with the use of ICD-10 codes for diagnosing hypertension, which may present issues with accuracy. Therefore, we conducted a sensitivity analysis focusing on cases where the diagnosis of hypertension was confirmed by both ICD-10 codes and the prescription of anti-hypertensive medications. However, in clinical practice, these drugs are sometimes prescribed for organ protection even in patients without a formal diagnosis of hypertension. Therefore, relying on prescription data alone does not fully guarantee the accuracy of hypertension diagnosis. To complement this, validation studies have assessed the accuracy of hypertension diagnoses based on ICD-10 codes in administrative claims data. For example, in a large cohort, diagnostic codes alone showed a sensitivity of 80.7%, specificity of 95.3%, positive predictive value of 84.3%, and negative predictive value of 94.1%, indicating reasonably high validity [20]. While combining diagnostic and medication codes further improves accuracy, these findings suggest that claims data using ICD-10 codes provide a relatively reliable method for identifying hypertension, although some misclassification may still remain.

In conclusion, our analyses of a nationwide epidemiological database suggest that lower LBM may be associated with a greater risk of developing hypertension in men. This highlights the potential clinical importance of the promotion of LBM through physical activity and dietary measures. Furthermore, from a future perspective, it is important to investigate whether supporting the increase of LBM as a health policy would be effective in reducing hypertension and its complications at the national level.

## Supplementary information

Supplementary information
