# Supplementary material for: Lean body mass index and hypertension risk in men: a nationwide epidemiological cohort study
Source: Hypertens Res. 2025 Nov 10;49(3):829–36. doi: 10.1038/s41440-025-02447-x (PMC12960225; doi:10.1038/s41440-025-02447-x)
Supplement: Supplementary file 1 — Supplementary information [file 41440_2025_2447_MOESM1_ESM.docx]

| **Supplementary Table S1. Association Between Body Mass Index and Hypertension Onset: Event Frequency, Incidence Rates, and Hazard Ratios.** | | | | | | |
| --- | --- | --- | --- | --- | --- | --- |
|  | Number | Event | Incidence Rate (95% CI) | Model 1 (Unadjusted) | Model 2 | Model 3 |
| Q1 | 96,138 | 7,159 | 243.7 (238.7–248.8) | 1 [Reference] | 1 [Reference] | 1 [Reference] |
| Q2 | 96,134 | 9,161 | 245.3 (240.4–250.4) | 1.28 (1.22–1.32) | 1.11 (1.07–1.15) | 1.10 (1.07–1.14) |
| Q3 | 96,141 | 10,812 | 271.8 (266.5–277.1) | 1.55 (1.51–1.60) | 1.22 (1.18–1.26) | 1.20 (1.17–1.25) |
| Q4 | 96,138 | 13,180 | 328.0 (322.1–333.9) | 1.98 (1.93–2.04) | 1.56 (1.50–1.62) | 1.50 (1.45–1.56) |
| The incidence rate was per 10,000 person-years. Unadjusted and adjusted hazard ratios (95% CI) for the development of hypertension are shown. Model 1 is unadjusted. Model 2 includes adjustment for age, systolic and diastolic blood pressures, and lean body mass index. Model 3 includes adjustment for age, systolic and diastolic blood pressures, lean body mass index, diabetes, dyslipidemia, cigarette smoking, alcohol drinking, and physical inactivity.  CI, confidence interval. | | | | | | |

| **Supplementary Table S2. Association Between Lean Mass Index and Hypertension Onset by Age: Event Frequency, Incidence Rates, and Hazard Ratios.** | | | | | | | |
| --- | --- | --- | --- | --- | --- | --- | --- |
|  |  | Number | Event | Incidence Rate (95% CI) | Model 1 (Unadjusted) | Model 2 | Model 3 |
| <65 years | Q1 | 61,691 | 3,920 | 152.5 (147.8–157.4) | 0.53 (0.51–0.55) | 1.24 (1.16–1.31) | 1.19 (1.12–1.26) |
|  | Q2 | 67,881 | 4,830 | 168.7 (164.0–173.5) | 0.59 (0.57–0.61) | 1.08 (1.02–1.13) | 1.06 (1.00–1.11) |
|  | Q3 | 72,169 | 6,230 | 208.6 (203.5–213.8) | 0.73 (0.70–0.75) | 1.07 (1.03–1.11) | 1.05 (1.01–1.10) |
|  | Q4 | 80,599 | 9,082 | 284.9 (279.1–290.8) | 1 [Reference] | 1 [Reference] | 1 [Reference] |
| ≥65 years | Q1 | 34,447 | 5,006 | 457.8 (445.3–470.7) | 0.73 (0.70–0.77) | 1.19 (1.10–1.29) | 1.14 (1.05–1.24) |
|  | Q2 | 28,259 | 4,404 | 488.8 (474.6–503.5) | 0.78 (0.75–0.82) | 1.08 (1.01–1.15) | 1.06 (0.99–1.13) |
|  | Q3 | 23,967 | 3,932 | 522.8 (506.8–539.4) | 0.84 (0.80–0.88) | 1.02 (0.97–1.08) | 1.01 (0.96–1.07) |
|  | Q4 | 15,538 | 2,908 | 620.9 (598.8–643.9) | 1 [Reference] | 1 [Reference] | 1 [Reference] |
| The incidence rate was per 10,000 person-years. Unadjusted and adjusted hazard ratios (95% CI) for the development of hypertension are shown. This subgroup analysis was conducted by stratifying participants based on age, using 65 years as the cutoff. Model 1 is unadjusted. Model 2 includes adjustment for age, systolic and diastolic blood pressures, and body mass index. Model 3 includes adjustment for age, systolic and diastolic blood pressures, body mass index, diabetes, dyslipidemia, cigarette smoking, alcohol drinking, and physical inactivity.  CI, confidence interval. | | | | | | | |

| **Supplementary Table S3. Association Between Lean Mass Index and Hypertension Onset in Non-obese Individuals: Event Frequency, Incidence Rates, and Hazard Ratios.** | | | | | | |
| --- | --- | --- | --- | --- | --- | --- |
|  | Number | Event | Incidence Rate (95% CI) | Model 1 (Unadjusted) | Model 2 | Model 3 |
| Q1 | 96,110 | 8,923 | 243.6 (238.6–248.7) | 1.16 (1.10–1.22) | 1.26 (1.18–1.35) | 1.17 (1.09–1.26) |
| Q2 | 95,581 | 9,140 | 243.9 (239.0–249.0) | 1.15 (1.10–1.22) | 1.09 (1.03–1.15) | 1.04 (0.98–1.11) |
| Q3 | 83,571 | 8,182 | 246.8 (241.6–252.3) | 1.17 (1.11–1.23) | 1.04 (0.98–1.09) | 1.01 (0.96–1.07) |
| Q4 | 18,627 | 1,617 | 211.7 (201.6–222.2) | 1 [Reference] | 1 [Reference] | 1 [Reference] |
| The incidence rate was per 10,000 person-years. Unadjusted and adjusted hazard ratios (95% CI) for the development of hypertension are shown. This sensitivity analysis focuses on individuals with a body mass index of less than 25.0 kg/m^2^, excluding those who are obese. Model 1 is unadjusted. Model 2 includes adjustment for age, systolic and diastolic blood pressures, and body mass index. Model 3 includes adjustment for age, systolic and diastolic blood pressures, body mass index, diabetes, dyslipidemia, cigarette smoking, alcohol drinking, and physical inactivity.  CI, confidence interval. | | | | | | |

| **Supplementary Table S4. Association Between Lean Mass Index and Hypertension Onset Defined by Both ICD-10 Code and Prescriptions for Antihypertensive Medication: Event Frequency, Incidence Rates, and Hazard Ratios.** | | | | | | |
| --- | --- | --- | --- | --- | --- | --- |
|  | Number | Event | Incidence Rate (95% CI) | Model 1 (Unadjusted) | Model 2 | Model 3 |
| Q1 | 93,077 | 5,865 | 163.5 (159.4–167.7) | 0.75 (0.73–0.78) | 1.32 (1.24–1.40) | 1.25 (1.18–1.32) |
| Q2 | 92,892 | 5,986 | 162.8 (158.7–167.0) | 0.75 (0.72–0.77) | 1.12 (1.06–1.17) | 1.09 (1.04–1.14) |
| Q3 | 92,560 | 6,586 | 180.6 (176.3–185.0) | 0.83 (0.80–0.86) | 1.06 (1.02–1.11) | 1.05 (1.00–1.09) |
| Q4 | 91,825 | 7,678 | 216.8 (212.0–221.7) | 1 [Reference] | 1 [Reference] | 1 [Reference] |
| The incidence rate was per 10,000 person-years. Unadjusted and adjusted hazard ratios (95% CI) for the development of hypertension are shown. This sensitivity analysis focuses on cases diagnosed with hypertension based on both ICD-10 codes and antihypertensive medication prescriptions. Model 1 is unadjusted. Model 2 includes adjustment for age, systolic and diastolic blood pressures, and body mass index. Model 3 includes adjustment for age, systolic and diastolic blood pressures, body mass index, diabetes, dyslipidemia, cigarette smoking, alcohol drinking, and physical inactivity.  CI, confidence interval. | | | | | | |

| **Supplementary Table S5. Association Between Lean Mass Index and Hypertension Onset after Multiple Imputations: Event Frequency, Incidence Rates, and Hazard Ratios.** | | | | | | |
| --- | --- | --- | --- | --- | --- | --- |
|  | Number | Event | Incidence Rate (95% CI) | Model 1 (Unadjusted) | Model 2 | Model 3 |
| Q1 | 124,913 | 11,511 | 241.4 (237.0–245.8) | 0.73 (0.71–0.75) | 1.24 (1.19–1.29) | 1.19 (1.14–1.24) |
| Q2 | 126,964 | 12,246 | 244.0 (239.7–248.4) | 0.74 (0.72–0.75) | 1.08 (1.04–1.11) | 1.06 (1.02–1.09) |
| Q3 | 127,067 | 13,554 | 270.4 (265.8–275.0) | 0.82 (0.80–0.84) | 1.03 (1.01–1.06) | 1.03 (0.99–1.05) |
| Q4 | 126,752 | 16,105 | 330.6 (325.5–335.7) | 1 [Reference] | 1 [Reference] | 1 [Reference] |
| The incidence rate was per 10,000 person-years. Unadjusted and adjusted hazard ratios (95% CI) for the development of hypertension are shown. Multiple imputation with chained equations to replace missing variables was employed and 505,696 individuals were involved in this sensitivity analysis. Model 1 is unadjusted. Model 2 includes adjustment for age, systolic and diastolic blood pressures, and body mass index. Model 3 includes adjustment for age, systolic and diastolic blood pressures, body mass index, diabetes, dyslipidemia, cigarette smoking, alcohol drinking, and physical inactivity.  CI, confidence interval. | | | | | | |

| **Supplementary Table S6. Association Between Lean Mass Index and Hypertension Onset in a Competing Risks Analysis: Event Frequency, Incidence Rates, and Hazard Ratios.** | | | | | | |
| --- | --- | --- | --- | --- | --- | --- |
|  | Number | Event | Incidence Rate (95% CI) | Model 1 (Unadjusted) | Model 2 | Model 3 |
| Q1 | 50,234 | 5,844 | 341.5 (332.9–350.4) | 0.85 (0.82–0.88) | 1.29 (1.21–1.38) | 1.23 (1.15–1.32) |
| Q2 | 45,333 | 5,340 | 347.5 (338.3–356.9) | 0.87 (0.84–0.91) | 1.13 (1.07–1.19) | 1.10 (1.04–1.16) |
| Q3 | 43,201 | 5,384 | 374.9 (365.0–385.0) | 0.95 (0.91–0.98) | 1.10 (1.05–1.15) | 1.08 (1.03–1.13) |
| Q4 | 40,507 | 5,074 | 394.1 (383.4–405.1) | 1 [Reference] | 1 [Reference] | 1 [Reference] |
| The incidence rate was per 10,000 person-years. Unadjusted and adjusted hazard ratios (95% CI) for the development of hypertension are shown. Fine-Gray subdistribution hazard model was employed and 179,275 individuals were involved in this sensitivity analysis. Model 1 is unadjusted. Model 2 includes adjustment for age, systolic and diastolic blood pressures, and body mass index. Model 3 includes adjustment for age, systolic and diastolic blood pressures, body mass index, diabetes, dyslipidemia, cigarette smoking, alcohol drinking, and physical inactivity.  CI, confidence interval. | | | | | | |
